# Recycling of Carbon Fibres and Subsequent Upcycling for the Production of 3D-CFRP Parts

**DOI:** 10.3390/ma15145052

**Published:** 2022-07-20

**Authors:** David Rabe, Eric Häntzsche, Chokri Cherif

**Affiliations:** Research Group “Textile Materials for Lightweight Construction”, Institute of Textile Machinery and High Performance Material Technology (ITM), Faculty of Mechanical Science and Engineering, Technische Universität Dresden, Helmholtzstr. 5, 01069 Dresden, Germany; eric.haentzsche@tu-dresden.de (E.H.); chokri.cherif@tu-dresden.de (C.C.)

**Keywords:** recycling, weft knitting, hybrid yarn, thermoplast, end of Life-CFRP

## Abstract

Carbon fibres (CF) are used in CF reinforced plastic (CFRP) components. However, waste from CF yarn trim, CFRP and the end of life (EOL) CFRP structures will cause a recycling challenge in the next decades because of strict environmental regulations. Currently, recycling is carried out almost entirely by the use of pyrolysis to regain CF as a valuable resource. This high temperature process is energy consuming, and the resulting fibres are brittle. Hence, they are not suitable for processing of textiles into yarns or new reinforcement structures. To enable grave to cradle processing, a new approach based on a solvolysis recovery of CF and subsequent yarn spinning to obtain hybrid yarns suitable for textile processing, especially by weft knitting, was the focus of the international research project IGF/CORNET 256EBR. For the first time, it was possible to process hybrid yarns made of rCF on a weft knitting machine to produce biaxial reinforced structures to form CFRP from recycled carbon fibres. Therefore, various modifications were done on the textile machinery. In this way, it was possible to process the rCF and to get out a reproducible textile structure for the production of 3D recycled CFRP (rCFRP) parts.

## 1. Introduction

Carbon fibres are the most commonly used high-performance fibres for mass-optimised, heavy-duty composites. However, the large amount of CF waste generated during the production of carbon fibre reinforced plastic and at the end of CFRP components’ lives pollutes the global environment. Therefore, the CFRP waste will cause socio-economic problems in the near future [1]. For this reason, the recovery of CF from CFRP and its reintroduction into the circular economy is a sustainable challenge considering the environmental impact and CO_2_ emissions [2]. Today, recovery is carried out almost exclusively by pyrolytic processes to regain the pure CF. In this process, the EOL CFRP components are exposed to high temperatures (450 to 600 °C) [1,3] to remove the entire surrounding thermoset matrix. The textile processing (e.g., carding, spinning and knitting) of the brittle CF obtained from pyrolysis is demanding and limits its application to downcycling into nonwoven or injection moulded pellets [4,5]. As a result, rCF composites are currently classified as low performance but high-cost products due to costly reprocessing [1]. A new approach is needed to meet existing sustainability requirements [2] and to obtain rCF that can be used for textile processing into hybrid yarns, and especially into textiles for the production of high-performance CFRP components.

The main objective of an international research project was the development and implementation of a new value chain consisting of CF recovery, re-sizing of the recycled CF (rCF) with a sizing to be developed and production of a hybrid yarn from the loose rCF. The rCF hybrid yarn serves as source material for further processing of the textile into innovative reinforcement structures for the production of recycled CFRP components.

At the Faculty of Textile Science and Technology of Shinshu University, a process for CFRP recycling was developed using superheated steam (<300 °C) and a suitable catalyst. In this solvolysis process, the epoxy matrix of the composite is decomposed into oily substances, and the CF can be recovered quickly and gently with a fibre length of up to 100 mm. Available technologies for the production of hybrid yarns have to be modified for the processing of rCF due to brittleness, lack of size of the smooth yarn surface and electrical conductivity. The results of the rCF-hybrid yarn development and production will follow in a separate publication.

Within the framework of the project, new hybrid yarn structures with scalable mechanical properties were developed at the ITM of the TU Dresden by modifying the DREF friction spinning for the rCF provided by Shinshu University. These rCF hybrid yarns were further processed for the first time into easily drapeable, semi-finished textile-reinforcement products using the multi-layer flat knitting technique. For this purpose, multi-layer flat knitting was specially adapted to the processing properties of the rCF hybrid yarns. The developed and manufactured semi-finished reinforcements were processed into thermoplastic CFRP components in a hot pressing process.

## 2. Materials and Methods

In the research project, the source material was the CF roving Toray T700SC 50C12k with a fineness of 800 tex. It was used as filament yarn (roving) for all tests. In the following, “rCF type 1” refers to untreated fibres, 100 mm in length, cut from this CF roving. “rCF type 1” corresponds to dry fibre production waste. “rCF type 3” refers to CF obtained from industrial waste, i.e., uncured or partially cured unidirectional (UD) CF tapes.

For this purpose, the industrial waste was cut into 100 mm × 100 mm pieces and subjected to solvolysis. The fibre recovery process was developed at the Faculty of Textile Science and Technology at Shinshu University [6,7]. In the first step, the surrounding thermoset matrix is carbonised under a protective inert gas atmosphere at 400 °C. In the second step, the remaining epoxy residues on the CF surface are removed under controlled atmospheric conditions (superheated steam, 400 °C, 0.3 MPa, short controlled dwell) by solvolysis. These two fibre-protecting recovery steps prevent the CF from losing their mechanical properties. In the third step of fibre recovery, all reaction residues are completely removed in a batch washing process with acetone. In the last step, the recovered rCF were recoated in a batch process with newly developed thermoplastic (PA6)-compatible titanium-oxide-based sizing.

For the rCF prepared in this way, a recycling process chain was developed and successfully implemented at the ITM of the TU Dresden, including the production of rCF hybrid yarns for semi-finished reinforcement products and thermoplastic composite components [8,9,10]. For the development of rCF hybrid yarns, the conventional yarn formation technology was used. For this purpose, the carding, drawing and spinning technologies were modified. For the optimal setting of the carding system with regard to roller speeds and distances, comprehensive investigations were carried out and the best possible carding parameters were derived. For the processing of the transverse force-sensitive rCF on the draw frame, further modifications were carried out, especially with regard to the regulation system. Two hybrid yarn structures, HY2 and HY6, were developed from the two different rCF types (rCF type 1 and 3) and processed into hybrid yarns on a DREF-3000 friction spinning machine that is available at ITM. In order to achieve a rCF mass fraction of 62% in the hybrid yarn, which corresponds to an rCF volume fraction of 50% in the later composite, a mass ratio of 90:10 (core to sheath) was set. The total fineness of the hybrid yarns produced was 600 tex.

**Figure 1:**
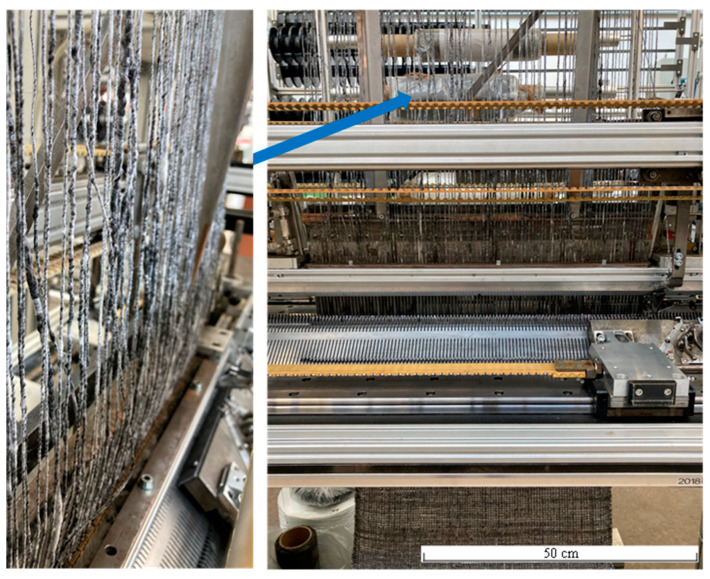
Modified biaxial weft knitting machine (gauge E5, working width 500 mm), with the warp yarn feed from the top of the machine (left picture, position shown by blue arrow).

For the realisation of biaxially reinforced rCFRP, the rCF hybrid yarns were processed for the first time on a modified flat knitting machine (Figure 1) into a 500 mm wide rCF weft knitted fabric (MLG) [11,12,13] with two reinforcing plies (consisting of a 90° weft and 0° warp ply) in order to comprehensively evaluate the textile processing properties.

**Figure 2:**
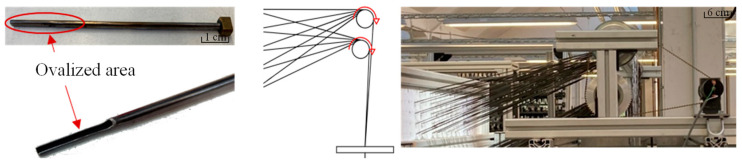
Adaptation measures on the biaxial flat knitting machine: (**left**) opening of the ovalized area of the warp yarn guides; (**right**) implementation of active self-regulating warp yarn delivery.

Due to the heterogeneous yarn characteristics (especially material-related imperfections in the yarn cross-section known as slubs), the knitting machine has been adapted in the area of the warp yarn feed. Due the openings of the metal warp, yarn guides were ovalized in the lower area because of the available space they were mechanically reworked (Figure 2, left) to reduce the otherwise occurring frictional forces. In order to compensate for the frictional forces on the yarn course of the warp thread feed from the creel to the needle bed (Figure 1), an active, self-regulating warp thread delivery (Figure 2, right) was realised. It consists of two driven rollers, 60 mm in diameter, that deflect the warp threads by approximately 90° to the needle bed. When a completed stitch row is pulled off at the needle bed, the frictional connection with the permanently rotating rollers increases. The warp yarn is conveyed until the frictional connection to the roller decreases due to the decreasing tensile force. With the help of these adjustments, it was possible to achieve good processability of the material.

**Figure 3:**
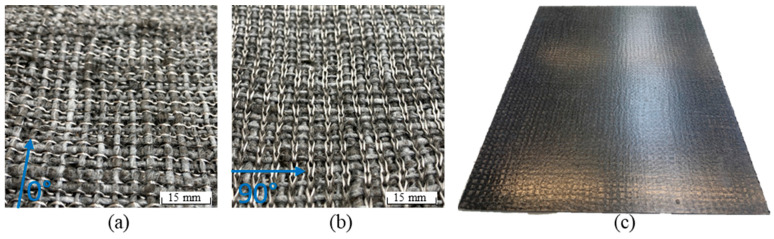
Weft knitted structure from the front (**a**) and the back (**b**) containing the rCF-hybrid yarn in weft (90°) and in warp (0°) directions, and hot-pressed into a (**c**) MLG-composite plates for mechanical characterisation.

Subsequently, the knitting depth was systematically varied (15, 15.5 and 16 mm) for low-damage rCF hybrid yarn due to the knitting process in order to achieve a balanced relationship between inherent stability and drapability of the MLG fabric. The well drapeable structures are able to be processed into composite components (with metal sheet-like forming behaviour) in a fast and cost-effective hot pressing process. The resulting reinforcement structure (see Figure 3) contains the rCF hybrid yarn in warp (0°) and weft directions (90°). The yarn systems are connected to each other by a hybrid stitch yarn consisting of a stretch-broken CF yarn (70 tex) and a PA6 filament yarn (94 tex). The structural property interactions of the link depth in the resulting textile structure (e.g., basis weight) and in the mechanical behaviour of the resulting composite structures (e.g., tensile and flexural strength) were investigated.

To characterise the composite properties, the rCF-MLG were processed into rCFRP composite plates by hot pressing. The pressing process (parameters: T = 295 °C; *p* = 4.2 MPa; t = 1200 s) ran in a vacuum chamber to get a composite with the minimum amount of entrapped air. Four (HY6) and eight (HY2) layers of the structure [0/90]_2,s_ resulted in a composite panel with 2 mm thickness (cf. Figure 3). After the pressing cycle, the mould could be taken out of the press; the plate could be demoulded and processed into specimens for the mechanical characterization. The processing of the composite plate was performed with a water-cooled diamond lab saw. The fibre volume content was measured by ashing of the composite and measuring the mass of the leftover fibres (DIN EN ISO 3451-1). Therefore, from every plate, three samples (25 mm × 10 mm) were cut out.

The panels were cut into six test specimens of 250 × 25 mm^2^ for every material variant for the characterisation of its tensile strength (DIN EN ISO 527-4). The composites made from the rCF weft knitted structures were tested in weft and warp directions. Using the results of the mechanical characterisation of the rCFRP made from the rCF-MLG, a preferred variant could be determined. 

Using the determined preferred variants of the rCF hybrid yarn, a rCF-MLG fabric was produced and processed into a “T-cup” demonstrator rCFRP component by hot pressing, similarly to metal sheet forming. For this purpose, eight layers of rCF-MLG were moulded in a hot press tool during the material-specific hot press cycle (cf. Table 1) with adapted heating time (t = 2400 s) due to the increased wall thickness. After completion of the process, the demonstrator component could be demoulded.

## 3. Results and Discussion

The investigations carried out have shown good machinability of rCF type 1 (new CF cut to 100 mm length) and rCF type 3 recovered by solvolysis using the modified DREF3000 friction yarn formation technique. Different rCF hybrid yarns (HY2, 6) were produced. The MLG flat knitting technology was adapted for processing rCF hybrid yarns into multilayer knitted fabrics (rCF-MLG). The modifications to the yarn guide resulted in good machine processability. The knitting depth was varied (15, 15.5 and 16 mm) in order to analyse the influences of this machine parameter on the textile and composite mechanical properties.

**Figure 4:**
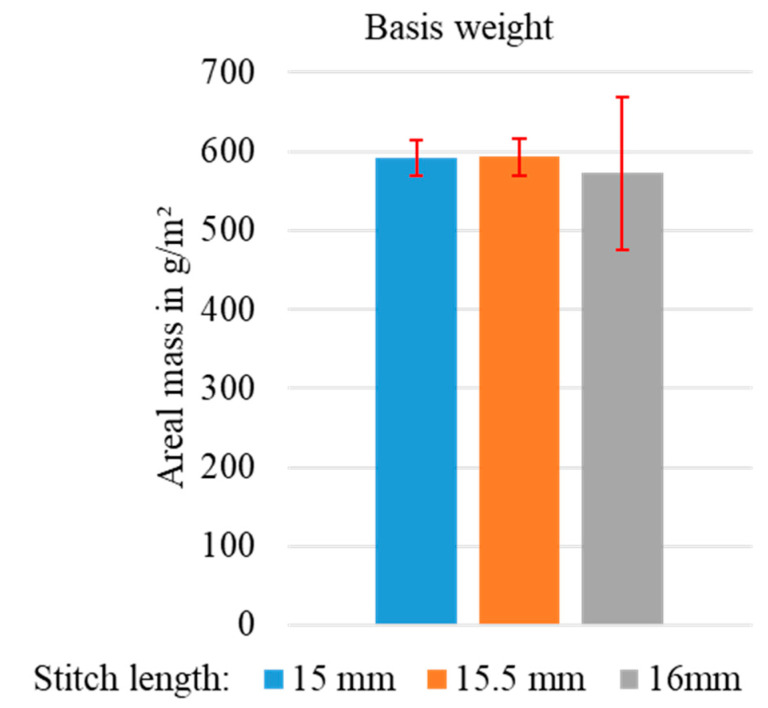
Basis weights of rCF multilayer weft knitted fabrics with dependence on stitch length.

The basis weights (cf. Figure 4) of the rCF-MLG with knitting depths of 15 and 15.5 mm show comparable values of approximately 600 g/m^2^. The mass per unit area of the sample with 16 mm knitting depth was lower due to the reduced weft density and showed higher variance of over 17%. In comparison, the variance of the variants with 15 and 15.5 mm knitting depth was less than 5%. The reason for this variance was the increased stitch length associated with the higher weft depth and the resulting reduced resistance to displacement of the textile structure.

**Figure 5:**
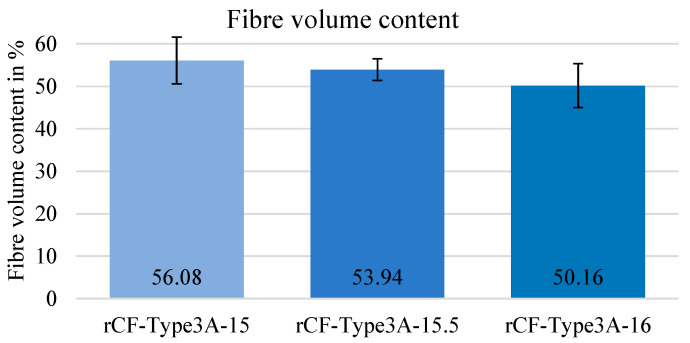
Measured fibre volume content by ashing using material made from HY6 (rCF-type 3), Indexation: rCF type 3 with sizing A (titanium oxide-based sizing)—stitch length in MLG (15/15.5/16 mm).

After the rCF-MLG made from HY 6 (rCF-Type 3) were processed into composites, the fibre volume content in relation to the stitch length was determined (cf. Figure 5). With a fibre volume content of more than 50% in each case, the measured values correspond to the previously calculated ones and hit the requirements of the project. With a higher knitting depth, the fibre volume content was lowered. The highest and so far best value was delivered for the composite structures made from rCF-MLG with a knitting depth of 15 mm.

**Figure 6:**
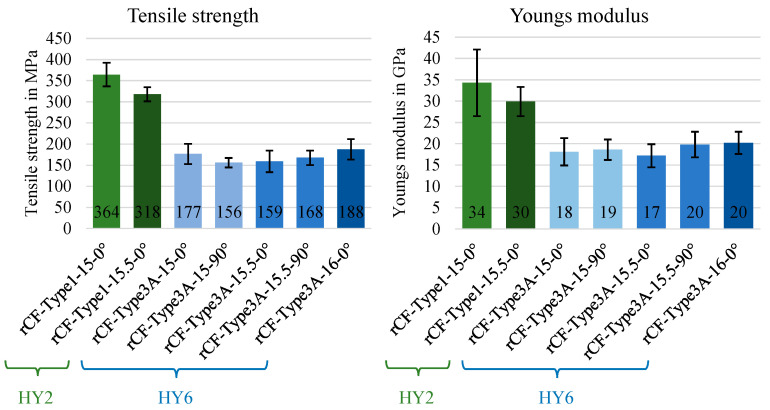
Tensile strength and Young’s modulus (DIN EN ISO 527-4) of rCFRP made of an rCF multi-layer weft knitted fabric. Indexation: rCF type in hybrid yarn (green: rCF-Type 1, blue: rCF-Type 3); HY—stitch length in MLG (15/15.5/16 mm)—test direction (0° warp/90° weft).

The mechanical behaviour was characterised in the tensile test (DIN EN ISO 527-4). Figure 6 shows the determined tensile strength and the Young’s modulus. The best mechanical properties were shown by the composites made of rCF-MLG with HY2 (rCF type 1) in the 0° or warp direction. The knitting depth of 15 mm was slightly advantageous in terms of mechanical properties compared to 15.5 mm. When comparing the values for tensile strength and Young’s modulus, it can be seen that the properties of the structures made of HY6 (rCF type 3) had about 50% lower values than those made of CF type 1. The influence of the knitting depth could not be significantly demonstrated and was definitely lower than the influence of the source yarn material.

By changing the stitch yarn, the resulting thickness of the reinforcement structure decreased, so instead of four, eight individual layers could be stacked to obtain a 2 mm thick sample plate. The small difference between 0° and 90° directions indicates comparable orthotropic material behaviour. The goal of obtaining comparable composite properties in the 0° and 90° directions was achieved. In order to increase the mechanical properties, further processing tests with rCF hybrid yarns with increased homogeneity are necessary.

**Figure 7:**
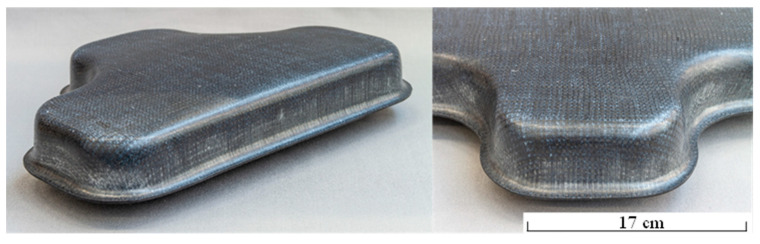
Realized 3D-rCFRP demonstrator “T-cup.”

The developed recycling process chain was successfully demonstrated by means of the 3D rCFRP component “T-cup” (Figure 7) made from rCF. Therefore, the preferred variant of rCF-MLG made from HY2 (rCF-Type 1, knitting depth: 15 mm) was used.

## 4. Conclusions

The recycling process chain developed enables full CFRP reuse in the form of high quality rCF in new thermoplastic composite components. At the same time, the resulting properties of the rCF hybrid yarns and the resulting textile reinforcement structures enable the rapid and highly productive production of 3D composite components from rCF for further applications, e.g., in automotive or plant engineering. The completed research project CORNET 265EBR contributes to overcoming the current disadvantages in the recycling of CF in a textile way, such as unsuitability of the brittle rCF for textile processing or non-aligned CF resulting in lower mechanical properties. The investigations have shown good CF recycling from EOL CFRP components. The hybrid yarn production by DREF3000 friction spinning was modified for the processing of rCF extracted from EOL CFRP by the developed two step recycling process. The resulting rCF hybrid yarns enable further textile technological processing into biaxially reinforced multilayer knits with aligned rCF. These can be converted into rCFRP components by means of a forming and downstream hot pressing process similar to metal sheet forming, which was successfully demonstrated using an application-oriented “T-cup” demonstrator, e.g., for the automotive sector. Future work will show even more information.

## Figures and Tables

**Table 1:** Parameters for pressing cycle of the demonstrator part “T-cup.”

Parameter	Value
Temperature T	295 °C
Pressure p	4.2 MPa
Time t	2400 s

## Data Availability

The data underlying this article will be shared on reasonable request from the corresponding author.
